# Patho-Ecological Distribution and Genetic Diversity of *Fusarium oxysporum* f. sp. *cubense* in Malbhog Banana Belts of Assam, India

**DOI:** 10.3390/jof11030195

**Published:** 2025-03-04

**Authors:** Anisha Baruah, Popy Bora, Thukkaram Damodaran, Bishal Saikia, Muthukumar Manoharan, Prakash Patil, Ashok Bhattacharyya, Ankita Saikia, Alok Kumar, Sangeeta Kumari, Juri Talukdar, Utpal Dey, Shenaz Sultana Ahmed, Naseema Rahman, Bharat Chandra Nath, Ruthy Tabing, Sandeep Kumar

**Affiliations:** 1 Biocontrol Laboratory, Department of Plant Pathology, Assam Agricultural University, Jorhat 785013, India; anisha.baruah.amj20@aau.ac.in (A.B.); saikiab605@gmail.com (B.S.); juri.talukdar@aau.ac.in (J.T.);; 2 ICAR-Central Institute of Subtropical Horticulture, Lucknow 226002, India; damhort73@gmail.com (T.D.);; 3 All India Coordinated Research Project on Fruits, ICAR-Indian Institute of Horticultural Research, Bengaluru 560089, India; 4 Krishi Vigyan Kendra, Sepahijala 799103, India; utpaldey86@gmail.com

**Keywords:** Fusarium wilt, Malbhog banana, India, diversity analysis, molecular characterization, polymorphism, race, AMOVA

## Abstract

Fusarium wilt, caused by *Fusarium oxysporum* f. sp. *cubense* (Foc), is recognized as one of the most devastating diseases affecting banana cultivation worldwide. In India, Foc extensively affects Malbhog banana (AAB genomic group) production. In this study, we isolated 25 Foc isolates from wilt-affected Malbhog plantations inIndia. A pathogenicity test confirmed the identity of these isolates as Foc, the primary causative agent of wilt in bananas. The morpho-cultural characterization of Foc isolates showed large variations in colony morphological features, intensity, and pattern of pigmentation, chlamydospores, and conidial size. The molecular identification of these isolates using Race1- and Race4-specific primers established their identity as Race1 of Foc, with the absence of Tropical Race 4 of Foc. For a more comprehensive understanding of the genetic diversity of Foc isolates, we employed ISSR molecular typing, which revealed five major clusters. About 96% of the diversity within the Foc population indicated the presence of polymorphic loci in individuals of a given population evident from the results of Nei’s genetic diversity, Shannon’s information index, and the polymorphism information content values, apart from the analysis of molecular variance (AMOVA). The current findings provide significant insights toward the detection of Foc variants and, consequently, the deployment of effective management practices to keep the possible epidemic development of disease under control along the Malbhog banana growing belts of northeast India.

## 1. Introduction

Bananas and plantains (*Musa* spp. Colla) belong to the order Zingiberales [1,2] and are one of the most widely traded fruit crops worldwide. They are extensively grown in tropical and subtropical areas [3,4,5] and hold a significant position in the global market, ranking alongside other major staple crops such as rice, wheat, and maize in terms of gross value [6,7]. Bananas hold a significant position as the fourth most important fruit crop globally after apples, grapes, and citrus fruits [8]. The origin of bananas can be traced back to the Pleistocene period, and their subsequent development is marked by the gradual domestication of local and export cultivars, predominantly the farmers of Southeast Asia [9]. Currently, bananas are cultivated in more than 150 countries worldwide, with the highest production (120 million tonnes) concentrated in Asia, Latin America, and Africa, whereas India and China are the largest producers in the domestic market [10]. The Indian banana industry holds a significant position in the global market, contributing 32% of the global basket of bananas, signifying the dominance of bananas in Indian horticulture. India’s annual export of bananas amounts to 1.35 million tonnes, worth USD 59.75 million. These bananas are primarily exported to major banana destinations such as Oman, Iran, Saudi Arabia, and the United Arab Emirates [11]. India is a significant player in the global banana market and holds a position as the second-largest producer of bananas worldwide, accounting for 20.1% of the global production. India cultivates bananas on an area of 0.80 million hectares, producing 29.7 million tonnes (MT)with a productivity of 34 metric tonnes/ha [12]. The banana industry of northeast India contributes 2.4% of the total Indian banana production [13], with Assam registering an annual production of 0.913 million tonnes of bananas from an area of 53.1 thousand hectares [14].

Northeast India, globally renowned for its banana diversity and being a national epicenter of banana production, is facing significant challenges due to the prevalence of Fusarium wilt [15]. The menace of this disease in this premier region assumes a more serious dimension, with favorable weather conditions for the proliferation of Foc, including heavy rainfall, allowing the spread of inoculums to newer areas [16], coupled with high humidity year-round, extended moisture availability in low-pH, coarse-textured soils [17], and chemical-free, low-input banana cultivation [13].

Improving our understanding of the progression of wilt disease in the field, adopting necessary cultural interventions, and eventually controlling the epidemic development of the pathogen have become increasingly formidable challenges for researchers due to the susceptibility of banana cultivars to infectious diseases caused by pathogenic microorganisms such as *Fusarium oxysporum* f. sp. *cubense* [18]. Malbhog banana (AAB genomic group) is a popular commercial cultivar, with the largest banana market located in Assam, India. The crop succumbs to several pests and diseases; among them, Fusarium wilt disease (also known as Panama wilt) poses an insurmountable risk to the banana industry with an upsurge in epidemics following the replacement of the susceptible Gros Michel banana variety with the Cavendish cultivar [19]. In amulti-locational trial conducted by the Indian Council of Agricultural Research (ICAR) underthe All-India Coordinated Research Project (ICAR-AICRP) on Fruits (2020), the incidence rates of this diseasewerereported to vary between 10 and 60%.

Wilt disease is a vascular disease caused by the soil-borne fungus *Fusarium oxysporum* f. sp. *cubense* (E.F. Smith) and transmitted primarily through the dissemination of infected plant material and long-lasting spores capable of surviving up to 20 years in the soil [20,21]. The identified causal agent attributed to Foc Race 1 has led to the eradication of the ‘Gros Michel’ banana cultivar [22,23]. Subsequently, the emergence of the novel Tropical Race 4 (TR4) of Foc has proven the susceptibility of the Cavendish subgroup to this devastating disease [24]. In India, Fusarium wilt disease was initially recorded in West Bengal, India. This reporting emphasizes the need for implementing more stringent quarantine measures to prevent the unwanted spread of the disease [25]. Recently, there has been a report of Foc TR4 in the Indian states of Uttar Pradesh [26] and Bihar [27], highlighting the increasing economic losses and consequential decline in banana production in the affected regions.

Foc isolates have been classified into four evolutionary physiological races based on their pathogenicity [28]. These isolates have the ability to infect plants belonging to the *Musaceae* (Banana plants) and *Heliconiaceae* (related plants) families. In fact, there are more than 100 formaspecialis of Fusarium wilt disease, each capable of affecting specific host plants in distinct ways [29,30]. Various methods, viz., vegetative compatibility group test [31,32,33,34], volatile aldehyde production analysis [35], molecular markers such as restriction fragment length polymorphism (RFLP) [36], Random Amplified Polymorphic DNA (RAPD) [37,38], and amplified fragment length polymorphism (AFLP) [39], have been extensively investigated for assessing the variability in races of the highly variable Foc pathogen [35]. In contrast, molecular markers serve as a powerful tool to analyze the genetic variation present within the Foc isolates from around the world [40]. However, the present scenario in India lacks sufficient data regarding the incidence and diversity of Foc in banana-growing regions.

The present study was carried out focusing on Malbhog banana growing belts in Assam, India, with the main objective of studying the genetic variation in the banana wilt-causing pathogen using patho-ecological and molecular variations to identify virulent strains for the effective management of Fusarium wilt disease.

## 2. Materials and Methods

### 2.1. Isolation, Characterization, and Maintenance of Isolates

A detailed survey was undertaken to assess the distribution of Fusarium wilt in the Malbhog banana growing belts of Assam, distributed over two major agro-climatic zones, viz., the lower Brahmaputra valley zone (Kokrajhar, Chirang, Barpeta, and Golpara) and the upper Brahmaputra valley zone (Jorhat) (Table 1 and Figure S1).

During the survey, plantations were selected based on their typical visual symptoms associated with Fusarium wilt. These symptoms comprised the yellowing and browning of leaves, starting from the older leaves coupled with upward progression, petiole breaking with a skirt-like appearance, splitting of the pseudostem at the base, and internal vascular discoloration of the pseudostem. An additional screening was also performed for oozing symptoms to rule out the possibility of bacterial wilt and damage caused by pseudostem weevil. The samples collected from 22 to 27 June 2022 were carefully packed in paper bags and labeled for further study at the Department of Plant Pathology, Assam Agricultural University, Jorhat, Assam (India).

Wilt-infected vascular strands were carefully excised from diseased psuedostems and cleaned; then, the lesions were sliced into thin sections using a sterilized blade. To avoid contamination, blades were sterilized with 0.5% sodium hypochlorite (NaOCl) for 2 min, followed by two washings with sterile distilled water to eliminate all possible traces of NaOCl.

The samples were first sterilized and then dried by wrapping them in Whatmann No. 1 filter paper. After this, 3–4 pre-treated sections were made from each sample and inoculated into Petri plates containing potato dextrose agar (PDA) media, which was supplemented with Streptomycin sulphate (1.2 mL/240 mL PDA) as an antibacterial agent. Samples were labeled and incubated at 28 ± 1 °C in a BOD-Incubator for 4 days until fungal mycelial growth was observed. The pathogens isolated were purified using hyphal-tip culture techniques and characterized. Subsequently, 25 cultures were maintained and recorded for colony characteristics, mycelial texture, topography, margin, and pigmentations. They were also characterized for morphological characters, viz., the size and shape of macro-and microconidia along with chlamydospores, and micro-photographed using a trinocular light microscope (Carl Zeiss, Axiocam, Jena, Germany) at a magnification of 400×.

### 2.2. Molecular Race Identification of Foc Isolates

The fungal genomic DNA was extracted using Himedia (HiPurA^TM^ Fungal DNA Purification kit, Kennett Square, PA, USA) following the instructions provided in the manual. DNA quality was assessed using spectrophotemetric estimation and quality-checked using A260/A280 values in the range of 1.8 to 2.0, which ensured high-quality genomic DNA. It was also checked on 0.8% agarose gel for reconfirmation of quality. Pathogen characterization was performed using fungal universal ITS sequencing using the ITS1-ITS4 primer combination using a PCR reaction and program as described by Damodaran et al. 2019 [26]. The molecular identity of the isolates was characterizedthrough amplification using universal ITS primers (ITS1 5′-TCCGTAGGTGAACCTGCGG-3′ and ITS4 (5′-TCCTCCGCTTATTGATATGC-3′), and sequence characterization was performed by double pass sequencing from both the forward and reverse directions. The data were analyzed by creating consensus using EMBOSS merger and further annotated using Basic Local Alignment Search Tool (BLAST) [41]. Furthermore, to rule out the possibility of non-pathogenic *Fusarium oxysporum* asas well as race identification, the isolates were characterized by sequencing PCR products amplified with Race 1- [41] and TR 4-specific primers [42].

### 2.3. Pathogenicity Assay of FusariumIsolates

Pathogenicity tests were conducted for the isolates using tissueculture (TC) banana plantlets (secondary hardened) of the variety Malbhog collected from ICAR-CISH, Lucknow, India. The experiment was conducted in a pot experiment using one-month-old plantlets with 3 replications for each isolate in a protected shade net house. Another set of 3 plantlets was maintained as a control for each of the isolates. To ensure a sterile environment, soil with specific characteristics (pH: 5.8, organic carbon: 3.2 g/kg) was filled in polypropylene bags and autoclaved at 121 °C for 15 min under 15 lb psi pressure for 5 consecutive days. The pots used for the experiment were sterilized in laminar airflow under UV light for 15–20 min for 3 consecutive days. Foc inoculum was prepared from a 5-day-old culture in Potato Dextrose broth (PDB) [43]. The cultures were incubated in a rotary shaker at 28 ± 1 °C for 5 days. Subsequently, the broth was filtered through double-layered muslin cloth to obtain the spore suspension, which was then diluted 50 times to achieve a concentration of 10^6^/mL. The number of spores was adjusted through a hemocytometer before inoculation. For the inoculation process, the root systems of Malbhog plantlets of uniform height were washed with running tap water, trimmed to one-third of their original root mass, and inoculated with 1000 mL of the conidial suspension (1 × 10^6^ conidia/mL) for 30 min. After inoculation, the plants were closely observed for symptom development after 30 days of inoculation. Another set of control plants was maintained by dipping the trimmed roots in sterilized water.

A pathogenicity test of the isolates was interpreted based on the following ratings for both leaf and vascular symptoms developed by the International Network for the Improvement of Banana and Plantains (INIBAP) [44].

Leaf symptoms index: 1—No streaking/yellowing of older leaves, plants appear healthy; 3—slight streaking or yellowing of older leaves; 5—streaking or yellowing on most of lower leaves; 7—extensive streaking or yellowing on all of the leaves; 9—dead/wilted plants. The ratings are the mean of three replicates.

Vascular discoloration rating: 1—Corm completely clean, no vascular discoloration; 3—isolated points of discoloration on vascular tissue; 5—discoloration of up to 1/3 of the vascular tissue; 7—discoloration of between 1/3 and 2/3 of the vascular tissue; 9—discoloration of greater than 2/3 of the vascular tissue; 11—total discoloration of the vascular tissue. These ratings included the mean of three replicates.

### 2.4. Analysis of Genetic Diversity Using ISSR Markers

The genetic diversity of 25 *Fusarium oxysporum* isolates was evaluated using 12 highly polymorphic ISSR (Inter Simple Sequence Repeat) primers with di- or tri-nucleotide repeats [45] (Table 2). PCR amplification was carried out with 2720 Thermal Cycler in 0.2 mL PCR tubes with a reaction volume of 10 μL, comprising the components (Emerald Amp^®^ PCR Master Mix, Takara, Shiga, Japan) with 50 ng template DNA and 50 picomoles of primer. The standard annealing temperature was fixed in the range of48–52 °C for 3 different sets of primers, and PCR amplification was repeated at least twice to achieve consistency in a banding pattern. The process was as follows: initial denaturation at 94 °C for 30 s, annealing for 40 s at 48 or 52 °C, extension for 1.30 min. at 72 °C, and final extension for 7 min. at 72 °C. The amplified products of PCR were detected by staining with 1 µLof EtBr (0.5 µL/mL) and visualized under a UV trans-illuminator (BIORAD, Molecular Imager Gel DOCTM XR, Hercules, CA, USA).

### 2.5. Data Analysis

The banding pattern observed in the agarose gel was scored as 1 or 0 for the occurrence or absenceof bands, and a binary matrix was generated. The data were analyzed in NTSYS PC 2.0 software to generate a dendrogram using the unweighted pair group method of the arithmetic mean (UPGMA) based on Jaccard’s similarity coefficients. The dataset was subsequently used for measuring genetic diversity among and within populations via parameters such as percentage of polymorphic loci, Nei’s gene diversity (h) [46], and Shannon’s information index (I) [47] using PopGene software version 1.32 [48]. The average values for Nei’s gene diversity index (h) and Shannon’s information index (I) were evaluated using GenAlex version 6.5 [49]. Additionally, the polymorphism information content (PIC) for dominant markers was calculated using the general equation

PIC=1−[f2+2(1−f2)]

where f is the marker frequency in the dataset. PIC assesses the discriminatory ability of a marker to detect polymorphism [50].

The genetic structure of Foc isolates was evaluated using analysis of molecular variance (AMOVA), which estimated the variance components and their statistically significant levels of variation between and within the 25 isolates, with GenAlex version 6.5. Genetic differentiation was determined using PhiPT, which calculated the level of genetic divergence in the samples. The test for statistical significance was performed with 9999 permutations of random shuffling.

## 3. Results

### 3.1. Wilt Symptomatology

**Figure 1:**
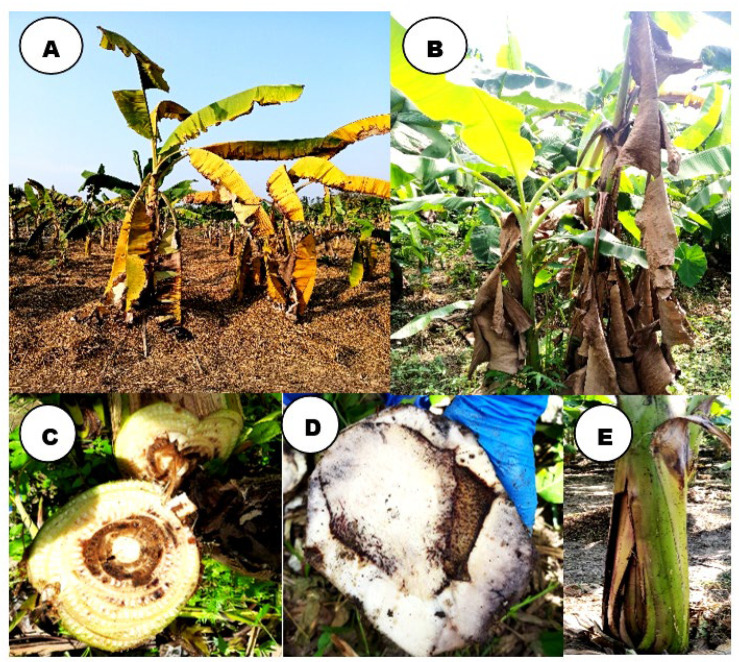
Typical external and internal wilting symptoms observed in Malbhog banana. (**A**) Yellowing and gradual wilting of older leaves, (**B**) petiole collapse and hanging giving a skirt-like appearance, (**C**,**D**) dark brown discoloration of vascular tissue, (**E**) splitting of pseudostem at the base.

Malbhog banana is extensively cultivated as one of the commercial cultivars in northeast India, which is affected by Fusarium wilt, and field diagnosis mainly relies on morphological symptoms. Typical symptoms of Fusarium wilt, such as the yellowing and gradual wilting of older leaves around the margins of leaf lamina, distortion of the leaf blade, and petiole collapse to collectively produce a hanging skirt-like appearance, were recorded (Figure 1). Eventually, all the infected plant leaves dried up, coupled with pseudostem splitting visible at the base, while the internal symptoms included the discoloration of infected rhizomes and pseudostems after examining either transverse or longitudinal sections. As the wilt infection advanced, the discoloration symptoms varied from pale-yellow to reddish-brown. These symptoms were recorded in more than 6-month-oldbanana plants. Many previous reports described these distinguishing characteristic symptoms depending on banana cultivars and growing conditions [51].

### 3.2. Isolation and Morphological Characterization of Foc Isolates

**Figure 2:**
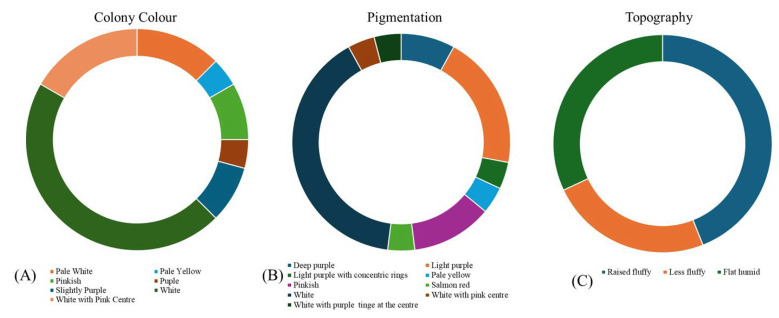
Graphical representation of the variation in cultural characteristics. (**A**): Colony color, (**B**): pigmentation, and (**C**): topography of isolated Foc isolates.

**Figure 3:**
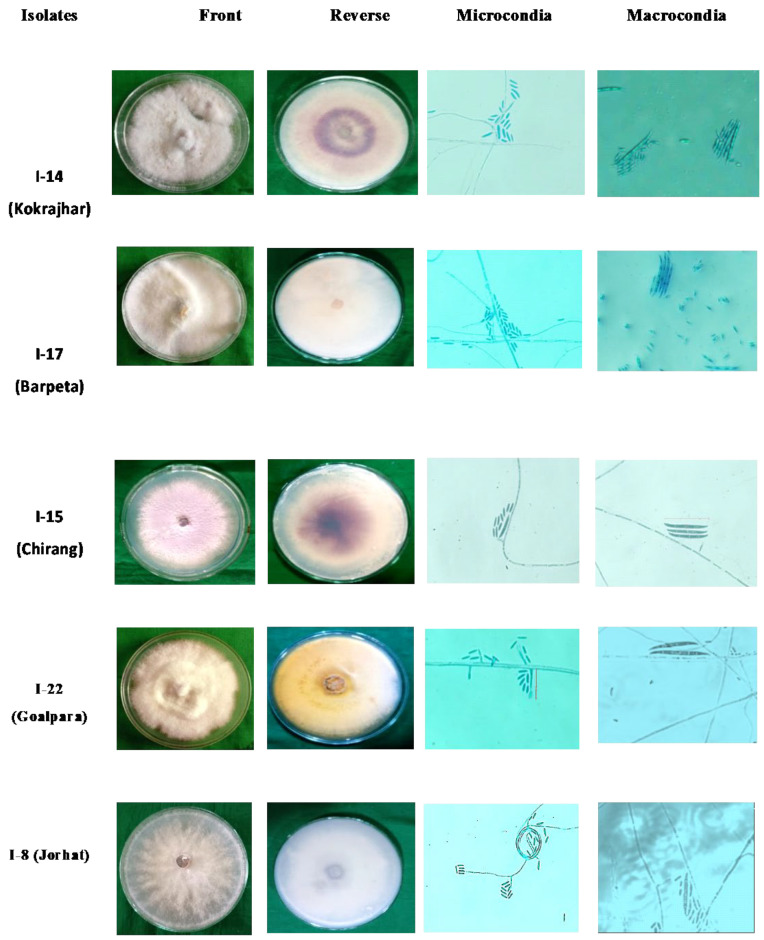
Morpho-cultural characteristics of five representative Foc isolates from five Malbhog banana belts of India.

The fungus was identified as *Fusarium oxysporum* using single-spore isolation on PDA and based on cultural (Table 3) and morphological characters (Table 4). Different Foc isolates were observed, displaying significant diversity in morpho-cultural characters, which described the colony color as white and white with pink pigmentation at the center. Among the 25 isolates, white was observed as the most dominant color (44%), followed by white with a pink center (18%), pale white (12%), pinkish (8%), slightly purple (8%), pale yellow (4%), and purple (4%) in a pattern of decreasing order (Figure 2). The shape of the colonies also showed little variability, as 23 isolates had circular smooth growth (92% of the isolates), and the remaining three displayed irregularly shaped mycelial growth (8% isolates) (Table 3). Concerning the mycelial texture of theFoc isolates, 11 isolates (44%) possessed abundant raised fluffy cottony mycelia, 6 isolates (24%) showed less fluffy hairy spaced mycelia, and the remaining 8 isolates (32%) showed flat humid mycelia (Figure 3). These Foc isolates exhibited great variability in their pigmentation on PDA media, where white-colored pigmentation was the most dominant color distributed in 11 isolates (44%), followed by a light purple color in 5 isolates (20%), a white purple center in 3 isolates (12%), deep purple pigmentation in 2 isolates (8%), light purple with concentric rings in 2 isolates (8%), salmon red pigmentation in 1 isolate (4%), and pale yellow in only 1 isolate (4%) (Figure 2). Previous studies reported varied colors and pigmentation patterns of Foc colonies from different banana belts, which comprised hairy to cottony mycelia, spaced or abundant, and variable from white to salmon to pale violet color [52]. It was also reported that some isolates tend to change rapidly from pionnotal (with abundant greasy or brilliant conidia aggregates) to flat humid mycelia of a white–pale yellowish to peach color on PDA culture [22,32].

A significant morphological variation among the Foc isolates was observed based on conidial (macro- and microconidia) size (Table 4 and Figure 3). The microconidia were typically oval-shaped cells with 0–2 septations and a size (µm) ranging from 10 to 12 length × 2.2 to 3.0 width. The maximum microconidia size (12 × 3.0 µm) was recorded with isolate 12 (I-12), while the lowest size (10 × 2.2 µm) was observed with isolate 17 (I-17). Likewise, macroconidia also showed great variability in size (27–30 × 3.2–3.6 µm) and septations (3–4), having a falcate shape with an attenuated tip and a foot basal cell. Maximum macroconidial size (30 × 3.6 µm) was observed with isolate 13 (I-13), while the lowest size (27 × 3.2 µm) was recorded with isolate 7 (I-7). The presence and absence of chlamydospores were observed as another distinguishing feature in the different isolates, which were typically round–oval–globose and developed singly or in chains, terminal or intercalary. The size of the chlamydospores ranged from 7.5 to 9.5 µm in diameter, with the maximum chlamydospore size (9.5 µm diameter) recorded for isolate 10 (I-10) and the minimum size (7.5 µm diameter) for isolate 2 (I-2). None of the isolates were observedto produce sporodochia.

### 3.3. Molecular Identification of Foc Isolates

**Figure 4:**
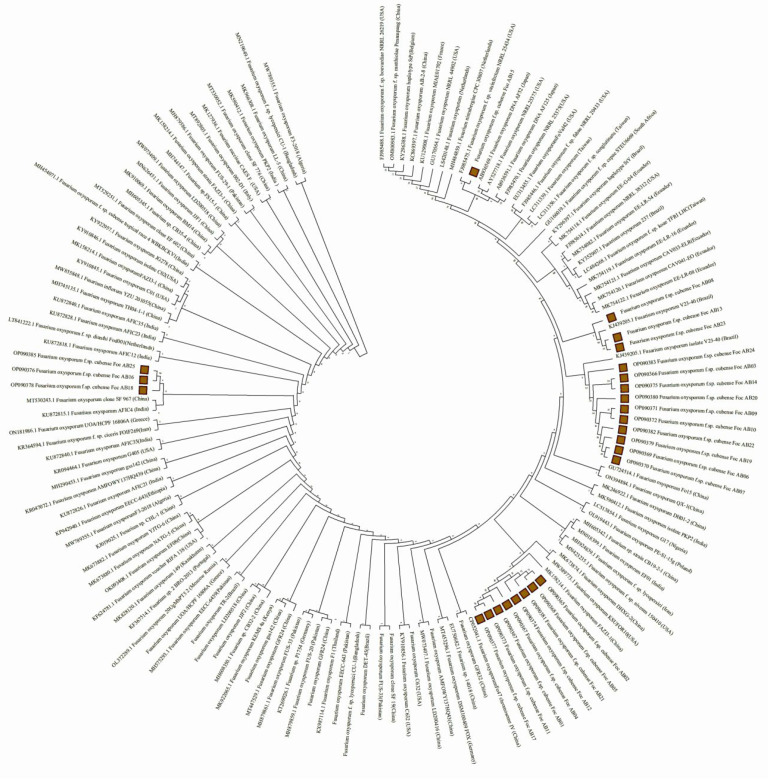
Radial phylogenetic tree of Foc isolates from Malbhog banana plantations using the maximum likelihood method, highlighting the 25 Foc isolates in red.

The high-quality genomic DNA of the 25 isolates was amplified with the Foc Race 1-specific primer to produce a ~354 bp band in agarose gel characteristic ofFoc. However, the TR4-specific primer did not produce any positive band at ~266 bp. Besides the race identification, preliminarily, the ITS-based identification of *Fusarium oxysporum* f. sp. cubense using the PCR products generated by the ITS1-ITS4 primer followed by sequencing and annotation by BLAST analysis resulted in identification to thespecies level [53]. The top hits of the BLAST analysis wereused for multiple sequence alignment and phylogenetic analysis using CLUSTAL software (http://www.clustal.org/clustal2/), which is shown as a radial tree in Figure 4. This phylogenetic tree is based on evolutionary relationships for various isolates following the maximum likelihood tree method with 500 bootstrap replicates. The results showed that Fusarium wilt fungus has the highest homology with *Fusarium oxysporum* (>98%). Considering both of the results, it is evident that all the isolates from the Malbhog banana belt were identified as FocRace 1.

### 3.4. Pathogenicity of Foc Isolates

**Figure 5:**
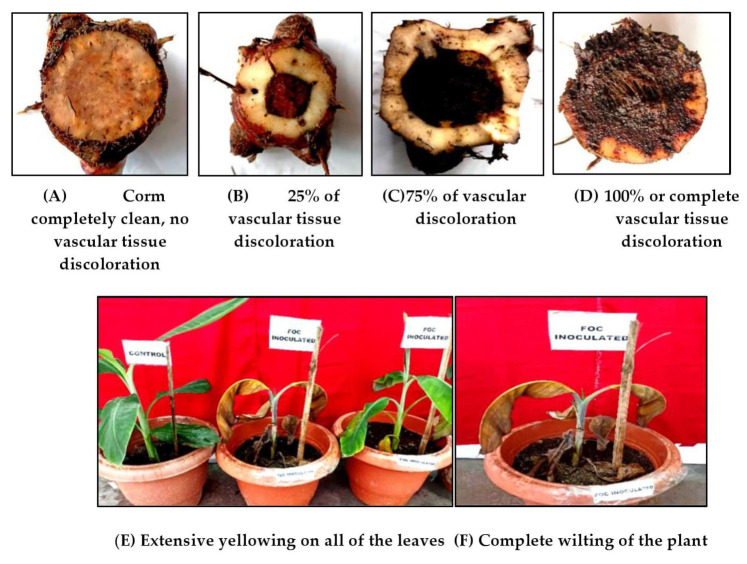
A representative picture showing the pathogenicity test of Foc isolates using TC Malbhog banana plantlets.

The pathogenicity test of the Foc isolates revealed the appearance of symptoms after 26 days to 33 days post-inoculation of the isolates (Table 5). The initial symptoms included the appearance of slight vein clearing, yellowing on the outer portion of the younger leaves followed by drooping of the leaves, stunting, and yellowing of the leaves. Defoliation and necrosis of the younger leaves occurred after 60 days of inoculation of the pathogen, and vascular discoloration of the corm was observed (Figure 5). Re-isolations were made from the inoculated Malbhog diseased plants on PDA medium, and the isolated fungus was purified and characterized for morphological and cultural features to confirm it as *Fusarium oxysporum* f. sp. cubenseRace 1.

### 3.5. Genetic Diversity Analysis Using ISSR Markers

**Figure 6:**
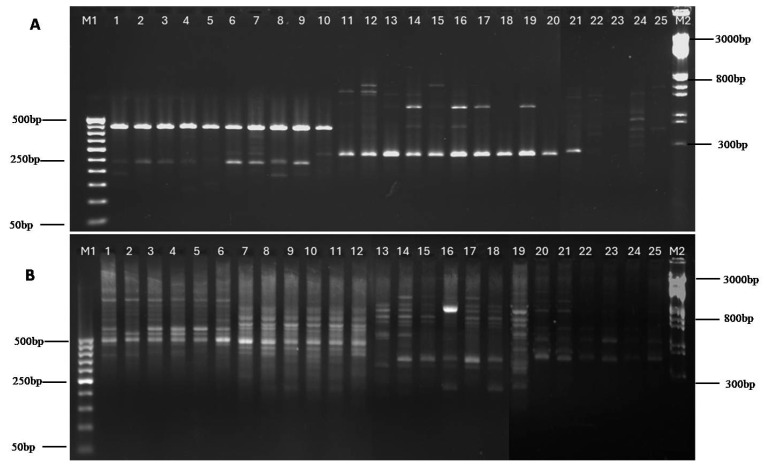
Representative DNA fingerprinting profiles of Foc isolates generated by 2 different ISSR molecular markers, viz., (**A**) (AC)8YG and (**B**) (AC)8G primers. The label at the top of the gel image indicates the following: M1—marker (100 bp ladder), 1 to 25—I-1 to I-25, M2—marker (1 kb ladder plus).

**Figure 7:**
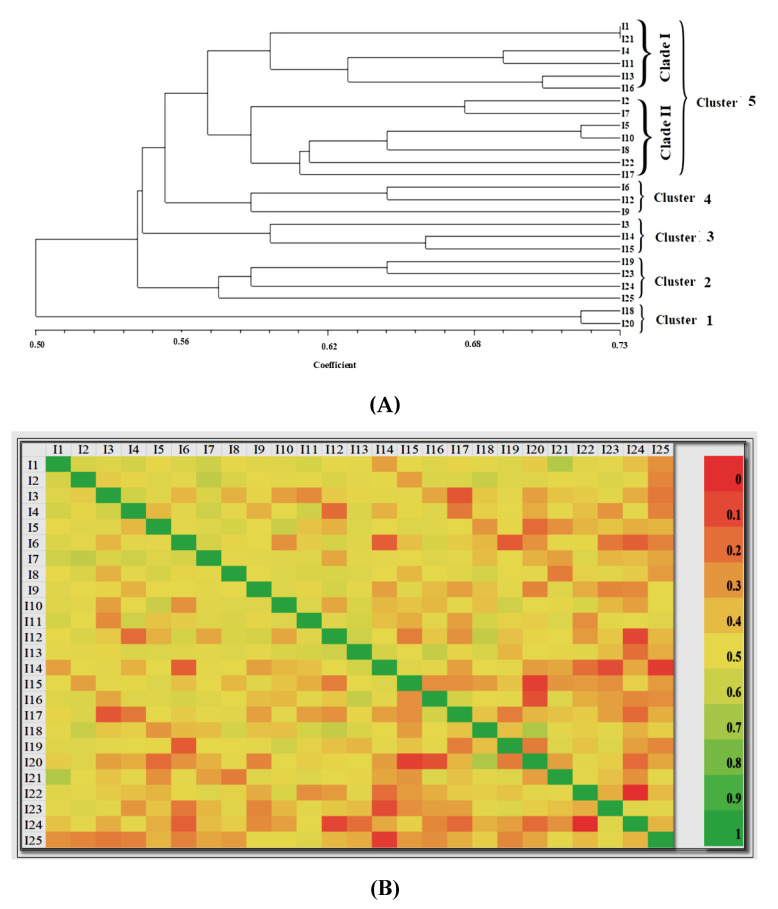
Dendrogram and heatmap of the 25 Foc isolates from the Malbhog belts of India. (**A**): Dendrogram derived from the UPGMA method using 12 ISSR markers, showing the genetic relationships among the 25 Foc isolates from the Malbhog belts of India. Five clusters were represented by Cluster 1 (I-18 and I-20); Cluster 2 (I-19, I-23, I-24, and I-25); Cluster 3 (I-3, I-14, and I-15); Cluster 4 (I-6, I-12, and I-9), and Cluster 5 (I-1, I-2, I-4, I-5, I-7, I-8, I-10, I-11, I-13, I-16, I-17, I-21, and I-22), indicating that Cluster 5 accommodates themaximum isolates. (**B**): Heat map showing Jaccard’s similarity coefficient matrix.

**Figure 8:**
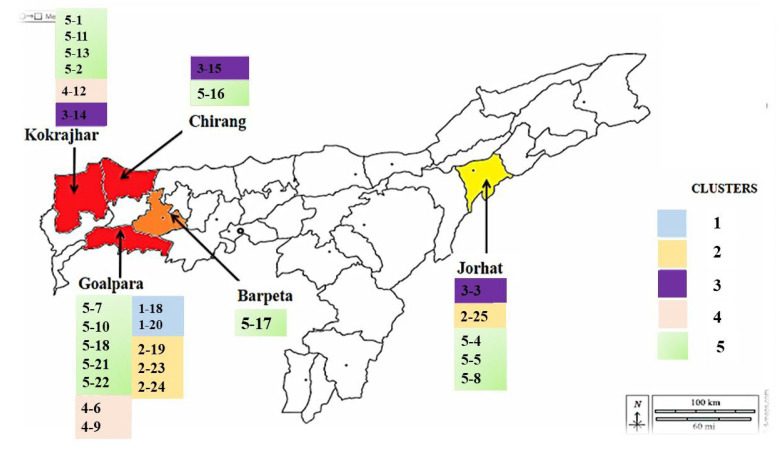
Distribution of Foc isolates from major Malbhog belts of Assam, India, using cluster-based analysis.

Foc isolates were subjected to genetic diversity analysis using 12 ISSR markers, which produced amplicons of 200 bp to 1 Kbp (Table 6 and Figure 6). A total of 4–8 bands were obtained for each isolate, and the presence of DNA bands was scored as 1 and 0 for the presence or absence of the bands, respectively, followed by studies on genetic interactions. The genetic diversity and polyphyletic nature among the 25 Foc isolates were observed using heat map analysis from the distance matrix developed from fingerprints of the Foc isolates derived from the Malbhog banana belt of India (Figure 7). The dendrogram generated using the UPGMA method grouped the 25 Foc isolates into five major clusters: 1, 2, 3, 4, and 5. The distribution pattern of the isolates in the clusters was 13 isolates in Cluster 5 (two sub-clusters, also called sub-clades, as clade 1 and clade 2 with a total of 13 isolates); 6 isolates in Clusters 3 and 4, each with 3 isolates; 4 isolates in Cluster 2; and 2 isolates in Cluster 1 (Figure 8). The isolates from Kokrajhar (I-1, I-11, I-13, I-2), Goalpara (I-7, I-10, I-18, I-21), Jorhat (I-4, I-5, I-8), Barpeta (I-17), and Chirang (I-16) showed maximum similarity under Cluster 5. Cluster 1(1–18, 1–20) belonged to Goalpara, and isolates from Cluster 2 belonged to Jorhat (I-25) and Goalpara (I-19, I-23, I-24). Cluster 3 isolates belonged to Kokrajhar (I-14), Chirang (I-15), and Jorhat (I-3). Similarly, isolates belonging to Cluster 4 belonged to Goalpara (I-6, I-9) and Kokrajhar (I-12) (Figure 8).

### 3.6. Population Structure

A total of 115 fragments were scored from the agarose gel profiles of 12 ISSR markers, among which 97% were polymorphic. Individual primers generated 5–8 bands, with an average of 5.2 bands. The number and percentages of polymorphic loci per primer (Table 6) showed the highest polymorphism of 100% for six primers. Nei’s genetic diversity (h) varied from 0.2600 to 0.4735, while Shannon’s index ranged from 0.3604 to 0.6660. Evidently, both the highest gene diversity (0.4735) and Shannon’s index (0.6660) were observed for primer (AC)8YA. The average values for Nei’s genetic diversity index (h) and Shannon’s information index (I) were recorded as 0.34 ± 0.010 and 0.50 ± 0.014, respectively. The high polymorphism and Nei’s genetic diversity (h) of 0.34 denoted comparatively higher levels of genetic diversity. In the current study, the average Shannon’s information index (I) was 0.50, denoting a moderate level of genetic diversity. The PIC value was estimated to evaluate the informative capacity of the primers. In the current study, the value ranged from 0.36 to 0.47 against the general maximum value of 0.5. High values of PIC suggest the effectiveness of the ISSR primers used in assessing genetic diversity. On average, a high polymorphic information content was observed for all the primers used. Primers (AC)8G, (AG)8C, and (GA)8YG showed the highest PIC values of 0.47, while (GA)8YT accounted for the lowest PIC of 0.36. AMOVA further confirmed the genetic variation in the Fusarium isolates. The AMOVA results further dividedthe overall variation into two levels, i.e., among populations and within populations (Table 7). A greater diversity to an extent of 96%was among the isolates within populations, indicating that polymorphic loci are prevalent in individuals of the given population. However, the lower amount of variation (4%) between populations showed that the populations are not highly divergent. The molecular variation among populations accounted for a meager 4%, while the variation within populations was 96%.

## 4. Discussion

Our study delved into themorphological characteristics of 25 isolates from Foc-infected Malbhog banana plants showing typical wilting and yellowing symptoms around the margins of the leaf lamina [54,55], distortion of the leaf blade, and petiole collapse producing a hanging skirt-like appearance [43,56] with the splitting of pseudostems at the base [57]. Internally, the infected rhizome and pseudostem showed discoloration [57,58,59]. These characteristic symptoms of Foc infection in banana plants have been documented in different studies [60,61]. The yellowing of leaves in Foc infection results from the release of fusaric acid by the pathogen, and wilting and chlorosis symptoms are reportedly due to the extensive formation of conidia in the xylem elements and blocking of vascular tissue [62,63]. Foc invades the root tissues of banana plants through natural openings, such as root hairs [41]. Upon invasion into the root, the pathogen colonizes and spreads through the xylem vessels, effectively blocking water and nutrient transport within the plant [64,65] and leading to defoliation and necrosis of younger leaves, as well as vascular discoloration in the corm. It has been reported that the production of cell wall-degrading enzymes, such as cellulases and pectinases, by the pathogen plays a critical role in the degradation of plant cell walls, allowing for successful penetration and colonization [66,67].

In order to understand the behavior and virulence of the pathogen, this article aimed to explore the fascinating realm of morpho-cultural and molecular diversity in Foc. In our observations, we found that 25 Foc isolates show different morphology, including colony color and shape. The morpho-cultural variability among the 25 isolates showed that the color of the mycelia was predominantly white (44%), followed by white with a pink center (18%), pinkish (8%), pale white (12%), slightly purple (8%), pale yellow (4%), and purple (4%). Further, variations were observed on the margins, and irregular or uniform circular-shaped and smooth margins were observed among different isolates. Based on the topography, flat humid, less fluffy hairy spaced, and abundant raised cottony fluffy characters were observed in the isolates. Most of the isolates had abundant aerial cotton mycelia (44%) as textural characters. The results of the present study are in consonance with the previous findings of earlier workers [32,35,68,69]. Another earlier study also found that pigmentation varied from white to pinkish dark purple and light purplish [40]. The variation in cultural and morphological characters may be due to the geographical locations of the isolates or it may be due to sudden heritable changes in the isolates. Rapid mutation from the pionnotal (with abundant greasy or brilliant conidia aggregates) to flat humid mycelia of a white–pale yellowish to peach color on a PDA culture was reported earlier [22,32].

In our study, the morphological variation among the isolates through macroconidia, microconidia, and chlamydospores also indicates significant variation among the isolates. The highest macroconidial size (30 × 3.6 µm) was recorded in isolateI-13, while the lowest size was recorded in isolate I-7 a t27 × 3.2 µm. The results of the morphological characterization in the present study are in agreement with those reported by several workers with reference to Foc [70,71], which also reported the size of the macroconidia, microconidia, and chlamydospores as being in the range obtained in the present investigation. In their findings, Perez-Vincente described macroconidia (27–55 × 3.3–5.5 µm) as abundant and falcate to erect to almost straight with thin walls with 3–5 septa (usually 3) and microconidia (5–16 × 2.4–3.5 µm) without septa, oval, elliptic to kidney-shaped, and develop abundantly in false heads in short monophialides [52]. The variation in macro- and microconidial size observed in Foc isolates highlights the importance of understanding the genetic diversity within this pathogen. This diversity may have implications for the pathogenicity and virulence of Foc, as well as its ability to adapt and evolve [72]. Previously, several studies were conducted to understand the pathogenicity of Foc and its effects on banana plants [73,74].

The pathogenicity test of our 25 isolates of Foc showed symptom development at 26–33 days after the inoculation of all the isolates, wherein isolates I-2, I-11, I-14, I-21, and I-24 showed the highest total vascular discoloration and the highest leaf symptoms index. Earlier, Aguilar-Hawod observed that isolates with a high vascular rating produced more aggressive leaf symptoms; however, race diagnosis via morphological characters was inconsistent [75]. These five isolates belonging to Kokrajhar (LBVZ) and Goalpara (LBVZ) also showed higher disease severity in the fields. This could be attributed to the mono-cropping of Malbhog banana in these areas [15], whichmight have augmented the virulence in these isolates besides congenial soil, climatic parameters, and inocula load. It was also observed that Foc Race 1 is commonly found in Assam due to various factors, including geographical location and environmental conditions [54]. Specifically, Race 1 was commonly found in tropical regions, such as the Philippines and Indonesia, which share similar climatic conditions with Assam. Assam, located in northeastern India, has a tropical climate characterized by high temperatures and humidity, providing favorable conditions for the survival and spread of Foc. These findings suggest that the virulence of Foc is not only influenced by genetic factors but also by environmental conditions specific to each geographical region [76].

The molecular identification of races within Foc is crucial for their accurate classification and the understanding of their genetic diversity. As a preliminary confirmation, the ITS-based sequence characterization identified all isolates to be *Fusarium oxysporum* isolates, and the nearest top hits of the BLAST analysis based on the constructed evolutionary phylogenetic tree clustered the isolates into three broad clusters. In the race identification of Foc isolates through race-specific PCR analysis, all the isolates invariably produced ~354 bp amplicon characteristics of Foc Race 1, confirming them to be Foc Race 1. Similar results have been reported with reference to race-specific primers developed by [41], which differentiated 11 Foc Race 1 isolates in China from Race 2.

Furthermore, studying genetic diversity is essential for understanding the evolutionary dynamics and population structure ofpathogens. Compared to theFoc isolates of Indian banana belts distributed across seven clusters identified through ISSR analysis [45], our diversity analysis in Malbhog banana belts revealed the presence of five different clusters, i.e., 1, 2, 3, 4, and 5. Of these clusters, the major cluster, i.e., Cluster 5, represented five districts, viz., Kokrajhar, Barpeta, Chirang, Goalpara, and Jorhat (I-1, I-2, I-4, I-5, I-7, I-8, I-10, I-11, I-13, I-16, I-17, I-21, and I-22), with two clades carrying the maximum isolates, followed by Cluster 4, Cluster 3, Cluster 2, and Cluster 1, in decreasing order of their dominance. Isolates (I-2) from Kokrajhar displayed maximum virulence, causing more than 76.00% percent disease incidence compared to the other isolates. The maximum diversity was observed in Goalpara district with four clusters (Clusters 1, 2, 4, and 5), followed by Kokrajhar (Clusters 3, 4, and 5) and Jorhat (Clusters 2, 3, and 5). Goalpara district has Asia’s largest banana market (Darangiri Banana market), and the growers rely on suckers from various nearby districts as planting materials. So, this may be a reason why Goalpara district shows the highest morpho-cultural and genetic variability among the isolates.

Jaccard’s coefficient similarity matrix further indicated that the isolates collected from the same location/region belonged to the same cluster; however, a few of them belonged to other clusters that are partially related to geographical regions, suggesting that these 25 Foc isolates are a mixture of isolates from the populations in our study. Such a possibility could be attributed to the collection and use of infected planting material from other geographical locations, or the pathogen diversity could be influenced by the agro-climate combination of the specific geographical location, its relationship with the crop, and the pathogen [77]. Chittarath reported that the isolates are uniquely associated with specific geographical regions [78]. However, there is limited information available on the biogeography of this genus in relation to soil and climate. Environmental factors such as rainfall, temperature, humidity, soil conditions, and local vegetation play a crucial role in determining the severity and spread of Fusarium species [79]. However, the foremost important factor is the relationship between the pathogen growth rate and temperature, besides the pathogen’s survival ability at extreme temperatures [80]. Fusarium spp. thrive inwarm and humid climates and at a temperature range of 24 to 30 °C [81,82,83,84]. High rainfall or excessive irrigation can create favorable conditions for the pathogen’s spores to germinate and infect the roots of susceptible Malbhog banana cultivars due to the development of more virulence and, thereby, the elevated disease incidence. More specifically, in India, the virulent strain of Race 1 has been reported inTN, AP, Gujarat, and Assam, whereas Foc TR4 has been reported in Bihar and U.P. [27,85]. Factors such as gene flow, spontaneous natural mutations, and genetic drift contribute to the variation in Foc isolates [86]. These reasons collectively contribute to the wide genetic diversity among Foc isolates with their polyphyletic nature. The genetic variation inFoc isolates studied by various researchers using different markers, such as RFLP, rDNA-ITS RFLP, and DNA [36,87], indicated wide genetic variation among Foc isolates. The clustering pattern using ISSR marker data in the present study also established that Foc isolates have clustered into five clusters as per the eco-geographical adaptations or location-specific clusters of Assam. The present study has clearly indicated that ISSR could precisely measure the genetic diversity among Foc isolates, as reported by earlier workers [88,89].

## 5. Conclusions

The diversity analysis of Foc isolates from Malbhog banana belts, as studied through morphological, cultural, and molecular tools, established the dominant presence of Race 1, a big respite to banana belts of this region. Studies relating to the evolutionary relationships of Foc isolates would further help in developing future management strategies. Therefore, the screening of Foc isolates from other commercial banana belts of India and the introduction of defense-related genes from wild banana germplasm as banana hot spots will become mandatory in order to develop a stringent strategy to contain the entry as well as the spread of Foc into newer banana belts. 

## Figures and Tables

**Table 1:** Details of geo-referenced sample collection sites representing Malbhog banana belts (agro-climatically, cultivation is confined to the lower and upper Brahmaputra valley zones, Assam) of India.

Sl. No.	Sample Code	Locations	Latitude	Longitude	PDI (%)
1.	I-1 (LBVZ)	Lakriguri, Gossaigaon, Assam, India	26.597429° N	89.927368° E	46.00
2.	I-2 (LBVZ)	Khoksaguri-II, Gossaigaon, Asam, India	26.50′76″96° N	89.898279° E	76.00
3.	I-3 (UBVZ)	Horticulture experimental Farm, AAU, Jorhat, Assam, India	26.726433° N	94.2046° E	18.00
4.	I-4 (UBVZ)	DakhinHatichungi, Jorhat, Assam, India	26.67404° N	94.190131° E	12.00
5.	I-5 (UBVZ)	Assam Agricultural University, Jorhat, Assam, India	26.726439° N	94.2021° E	20.00
6.	I-6 (LBVZ)	Dudhnoi, Assam, India	25.975957° N	90.81605° E	31.00
7.	I-7 (LBVZ)	Fafal, Dudhnoi, Assam, India	25.97548° N	90.815983° E	54.00
8.	I-8 (UBVZ)	Gohain Gaon, Jorhat, Assam, India	26.481931° N	94.162094° E	17.00
9.	I-9 (LBVZ)	Dhanubhanga, Goalpara, Assam, India	25.972059° N	90.980104° E	35.00
10.	I-10 (LBVZ)	Mandangpt-II, Assam, India	25.940924° N	90.966416° E	29.00
11.	I-11(LBVZ)	Bajugaon, No.1, Gossaigaon, Assam, India	26.491766° N	89.899861° E	64.00
12.	I-12 (LBVZ)	Bajugaon, No.1, Gossaigaon, Assam, India	26.490674° N	89.899704° E	38.00
13.	I-13 (LBVZ)	Bajugaon, No.2, Gossaigaon, Assam, India	26.495045° N	89.903236° E	39.00
14.	I-14 (LBVZ)	Bajugaon, No.3, Gossaigaon, Assam, India	26.496353° N	89.902717° E	46.00
15.	I-15 (LBVZ)	Chirang, Assam, India	26.545864° N	90.548317° E	29.00
16.	I-16 (LBVZ)	Chirang, Assam, India	26.555215° N	90.527732° E	38.00
17.	I-17 (LBVZ)	Sulikata, Barpeta, Assam, India	26.556428° N	90.848494° E	26.00
18.	I-18 (LBVZ)	Dahela, Goalpara, Assam, India	26.030963° N	90.74946° E	22.00
19.	I-19 (LBVZ)	Karkashi, Goalpara, Assam, India	26.029343° N	90.74946° E	31.00
20.	I-20 (LBVZ)	Dahela, Goalpara, Assam, India	26.033461° N	90.747591° E	47.00
21.	I-21(LBVZ)	Karkashi, Goalpara, Assam, India	26.034834° N	90.737234° E	19.00
22.	I-22 (LBVZ)	Kharamedhipara, Goalpara, Assam, India	26.001823° N	90.794572° E	41.00
23.	I-23 (LBVZ)	Kharamedhipara, Assam, India	26.001841° N	90.794601° E	30.00
24.	I-24 (LBVZ)	Mandang, Goalpara, Assam, India	25.941226° N	90.966125° E	27.00
25.	I-25 (UBVZ)	AAU, Jorhat, Assam, India	26.726433° N	94.2046° E	8.00

LBVZ and UBVZ represent the lower Brahmaputra valley zone and the upper Brahmaputra valley zone, respectively. PDI: percent disease incidence (%).

**Table 2:** Details of ISSR primers used to differentiate *Fusarium* isolates from Malbhog banana belts of India.

Sl. No.	Primers	Sequence (5′-3′)	Primer Length (bp)	Annealing Temperature (°C)
1.	(AC)8YA	ACACACACACACACACYA	18	50
2.	(AC)8G	ACACACACACACACACG	17	50
3.	(GA)8YC	GAGAGAGAGAGAGAGAYC	18	50
4.	(AG)8T	AGAGAGAGAGAGAGAGT	17	50
5.	(GA)8YT	GAGAGAGAGAGAGAGAYT	18	52
6.	(GA)8YT	AGAGAGAGAGAGAGAGYT	18	52
7.	(CA)8T	CACACACACACACACAT	17	52
8.	(AG)8C	AGAGAGAGAGAGAGAGC	18	52
9.	(CA)8RG	CACACACACACACACARG	18	48
10.	(GA)8YG	GAGAGAGAGAGAGAGAYG	18	48
11.	(CA)8RC	CACACACACACACACARC	18	48
12.	(AC)8YG	ACACACACACACACACYG	18	48

**Table 3:** Cultural traits of 25 *Fusarium* isolates obtained from Malbhog banana belts of India.

Sl. No.	Isolates	Colony Color	Mycelial Texture	Margin	Shape	Pigmentation
1.	I-1	White	Abundant Raised fluffy cottony	Smooth	Circular	White
2.	I-2	White with pink center	Flat humid	Smooth	Circular	Light purple
3.	I-3	Light purple	Raised fluffy	Smooth	Circular	Deep purple
4.	I-4	White	Flat humid	Irregular	Circular	White
5.	I-5	White with pink center	Raised fluffy	Smooth	Circular	Light purple
6.	I-6	Pinkish	Less fluffy	Smooth	Irregular	Light purple
7.	I-7	White	Flat humid	Smooth	Circular	White
8.	I-8	Pale white	Flat humid	Smooth	Circular	White
9.	I-9	Purple	Flat humid	Smooth	Irregular	Deep purple
10.	I-10	White with pink center	Raised fluffy	Smooth	Circular	Light purple
11.	I-11	White	Flat humid	Smooth	Circular	White with purple center
12.	I-12	White	Less fluffy	Irregular	Circular	White with purple center
13.	I-13	Pinkish	Raised fluffy	Smooth	Irregular	Salmon red
14.	I-14	White	Raised fluffy	Smooth	Circular	Light purple with concentric rings
15.	I-15	Slightly purple	Less fluffy	Smooth	Circular	Light purple
16.	I-16	White	Less fluffy	Irregular	Circular	White
17.	I-17	Pale white	Less fluffy	Smooth	Circular	White
18.	I-18	White	Raised fluffy	Smooth	Circular	White with purple center
19.	I-19	White with pink center	Flat humid	Smooth	Irregular	Light purple with concentric rings
20.	I-20	White	Raised fluffy	Smooth	Circular	White
21.	I-21	Pale white	Flat humid	Smooth	Circular	White
22.	I-22	Pale yellow	Less fluffy	Smooth	Circular	Pale yellow
23.	I-23	White	Raised fluffy	Smooth	Circular	White
24.	I-24	White	Raised fluffy	Smooth	Circular	White
25.	I-25	White	Raised fluffy	Smooth	Circular	White

**Table 4:** Morphological characteristics of 25 Fusarium isolates obtained from Malbhog banana belts of India.

Foc Isolates	Microconidia *			Macroconidia *			Chlamydospores	
	Size (µm)	No. of Septations	Shape	Size (µm)	No. of Septations	Shape	Size (µm)	Shape
I-1	10 × 2.5	1	Oval	28 × 3.4	3	Falcate	7.7	Oval
I-2	10 × 2.4	0	Oval	28 × 3.4	3	Falcate	7.5	Oval
I-3	11 × 2.5	0	Oval	30 × 3.5	3	Falcate	8.2	Round
I-4	11 × 2.4	2	Oval	30 × 3.3	3	Falcate	8.3	Oval
I-5	12 × 2.5	1	Oval	29 × 3.5	3	Falcate	9.0	Round
I-6	11 × 2.6	1	Oval	28 × 3.6	3	Falcate	8.5	Round
I-7	11 × 2.8	1	Oval	27 × 3.2	3	Falcate	8.0	Round
I-8	11 × 3.0	1	Oval	30 × 3.5	3	Falcate	9.0	Oval
I-9	11 × 2.6	2	Oval	29 × 3.5	3	Falcate	8.8	Oval
I-10	11 × 3.0	0	Oval	28 × 3.2	3	Falcate	9.5	Round
I-11	11 × 2.4	1	Oval	27 × 3.4	3	Falcate	8.1	Oval
I-12	12 × 3.0	0	Oval	30 × 3.5	3	Falcate	8.5	Oval
I-13	11 × 3.0	1	Oval	30 × 3.6	3	Falcate	9.0	Oval
I-14	12 × 2.4	1	Oval	28 × 3.5	3	Falcate	8.7	Round
I-15	12 × 2.5	2	Oval	28 × 3.5	3	Falcate	8.9	Oval
I-16	11 × 2.5	1	Oval	29 × 3.4	3	Falcate	9.1	Oval
I-17	10 × 2.2	0	Oval	29 × 3.5	3	Falcate	9.2	Oval
I-18	12 × 2.5	1	Oval	27 × 3.6	3	Falcate	8.1	Round
I-19	11 × 3.0	2	Oval	30 × 3.4	3	Falcate	8.6	Round
I-20	11 × 2.6	1	Oval	28 × 3.5	4	Falcate	8.2	Oval
I-21	10 × 2.5	0	Oval	30 × 3.6	3	Falcate	9.0	Oval
I-22	10 × 2.4	0	Oval	30 × 3.5	3	Falcate	9.1	Round
I-23	11 × 2.5	1	Oval	29 × 3.5	3	Falcate	8.3	Oval
I-24	12 × 2.5	1	Oval	29 × 3.4	4	Falcate	8.7	Round
I-25	11 × 3.0	1	Oval	28 × 3.5	3	Falcate	9.0	Oval

* data are the mean of 5.

**Table 5:** Pathogenicity of Foc isolates using tissue culture from Malbhogbanana.

Sl. No	Isolate Code	Days After First Appearance of the Symptoms	Leaf Symptoms Index	Vascular Discoloration Rating
1.	I-1	26	7	7
2.	I-2	27	9	11
3.	I-3	30	7	9
4.	I-4	33	9	9
5.	I-5	27	7	7
6.	I-6	26	5	5
7.	I-7	32	9	9
8.	I-8	29	7	7
9.	I-9	30	3	3
10.	I-10	30	5	5
11.	I-11	28	9	11
12.	I-12	31	3	3
13.	I-13	26	9	9
14.	I-14	28	9	11
15.	I-15	32	5	5
16.	I-16	29	3	3
17.	I-17	33	7	7
18.	I-18	27	7	7
19.	I-19	29	3	3
20.	I-20	30	5	5
21.	I-21	32	9	11
22.	I-22	33	5	9
23.	I-23	29	5	5
24.	I-24	26	9	11
25.	I-25	27	5	5

**Table 6:** Genetic diversity estimates of the 12 ISSR primers used for polymorphism.

Primer	Number of PL *	Percentage of PL (%)	PIC †	h **	I ††
(AC)8YA	25	100	0.41	0.4735	0.6660
(AC)8G	25	100	0.47	0.4672	0.6592
(GA)8YC	25	100	0.43	0.4480	0.6385
(AG)8T	25	100	0.43	0.4288	0.6178
(GA)8YT	24	96	0.36	0.3904	0.5701
(AG)8YT	13	52	0.44	0.2600	0.3604
(CA)8T	23	92	0.44	0.4160	0.5916
(AG)8C	24	96	0.47	0.4333	0.6166
(CA)8RG	22	88	0.40	0.3700	0.5367
(GA)8YG	25	100	0.47	0.4675	0.6596
(CA)8RC	25	100	0.39	0.4032	0.5902
(AC)8YG	24	96.00	0.42	0.4224	0.6047

* PL = polymorphic loci, † PIC = polymorphism information content, ** h = Nei’s gene diversity, †† I = Shannon’s information index.

**Table 7:** Summary of the analysis of molecular variance (AMOVA) for 25 isolates of *Fusarium oxysporum* f. sp. *cubense*.

Source	Degrees of Freedom (df)	Sum of Squares	Mean Square	Estimated Variance	% Variation	*p*-Value
Among Pops	4	65.520	16.380	0.608	4%	<0.001
Within Pops	20	266.800	13.340	13.340	96%	<0.001
Total	24	332.320	29.72	13.948	100%	<0.001

## Data Availability

All the sequences of Foc isolates were submitted to NCBI, and GenBank accessions are supplemented as Supplementary Table (Table S1).

## Supplementary Materials

The following supporting information can be downloaded at https://www.mdpi.com/article/10.3390/jof11030195/s1.
