# Supplementary material for: Patho-Ecological Distribution and Genetic Diversity of Fusarium oxysporum f. sp. cubense in Malbhog Banana Belts of Assam, India
Source: J Fungi (Basel). 2025 Mar 4;11(3):195. doi: 10.3390/jof11030195 (PMC11942760; doi:10.3390/jof11030195)
Supplement: Supplementary file 1 [file jof-11-00195-s001.zip › jof-3232759-supplementary.pdf]

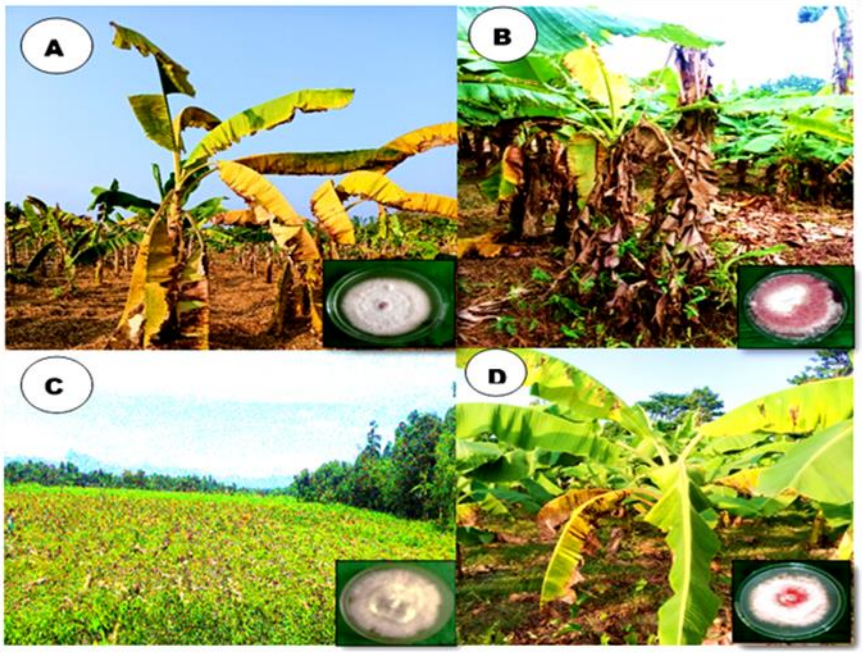

Figure S1: Field view of surveyed locations of Malbhog banana plantations infected with Fusarium wilt: (A) Jorhat, (B) Barpeta, (C) Gossaigaon, (D) Goalpara. The variation in field symptoms of banana wilt could be easily observed.

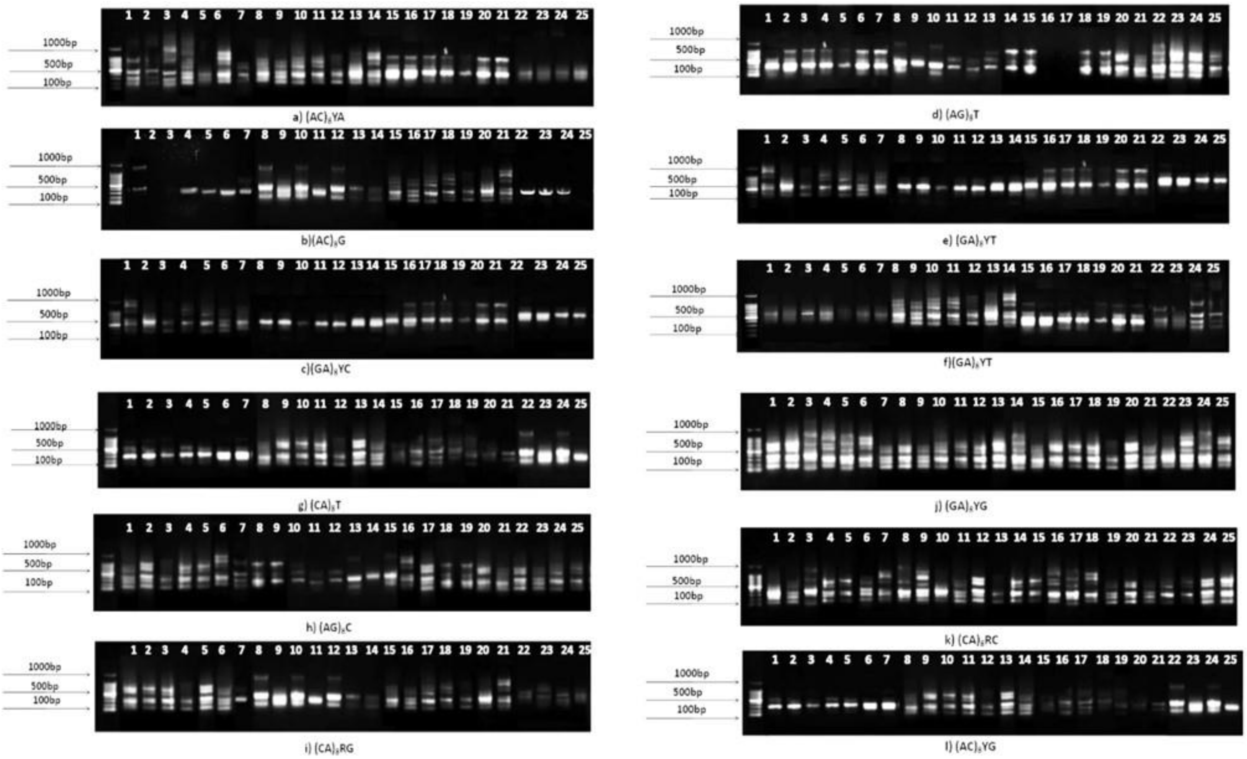

Figure S2 Representative DNA fingerprinting profiles of Foc isolates generated by 12 different ISSR molecular markers.

Table S1: List of Accession numbers of different *Fusarium oxysporum* f.sp. *cubense* isolates in GenBank record, NCBI

Sl. No.	Isolate	Accession number
1	I-1	OP090364 Foc_AB01
2	I-2	OP090365 Foc_AB02
3	I-3	OP090366 Foc_AB03
4	I-4	OP090367 Foc_AB04
5	I-5	OP090368 Foc_AB05
6	I-6	OP090369 Foc_AB06
7	I-7	OP090370 Foc_AB07
8	I-8	OP090756Foc_AB08
9	I-9	OP090371 Foc_AB09
10	I-10	OP090372 Foc_AB10
11	I-11	OP090373 Foc_AB11
12	I-12	OP090374 Foc_AB12
13	I-13	OP090651 Foc_AB13
14	I-14	OP090375 Foc_AB14
15	I-15	OP090755Foc_AB15
16	I-16	OP090376 Foc_AB16
17	I-17	OP090377 Foc_AB17
18	I-18	OP090378 Foc_AB18
19	I-19	OP090379 Foc_AB19
20	I-20	OP090380 Foc_AB20
21	I-21	OP090381 Foc_AB21
22	I-22	OP090382 Foc_AB22
23	I-23	OP090652 Foc_AB23

24	I-24	OP090383 Foc_AB24
25	I-25	OP090384 Foc_AB25
